# Bis{1-[2-(diphenyl­phosphan­yl)eth­yl]-3-ethyl­imidazol-2-yl­idene}nickel(II) diiodide acetonitrile disolvate

**DOI:** 10.1107/S1600536812028784

**Published:** 2012-06-30

**Authors:** Aziza Ahmida, Domenica Withake, Ulrich Flörke, Hans Egold, Gerald Henkel

**Affiliations:** a Department Chemie, Fakultät für Naturwissenschaften, Universität Paderborn, Warburgerstrasse 100, D-33098 Paderborn, Germany

## Abstract

The mol­ecular structure of the title compound, [Ni(C_19_H_21_N_2_P)_2_]I_2_·2CH_3_CN, shows two six-membered *N*-heterocyclic carbene/phosphane chelate rings that form a nearly square-planar coordination geometry around the Ni^II^ atom, which lies 0.190 (1) Å above the C_2_P_2_ plane. The sum of the bond angles at the Ni^II^ atom is 358.68 (6)°, with C—Ni—P bite angles of 82.89 (5) and 84.08 (6)°. The two carbene rings make a dihedral angle of 52.65 (8)°.

## Related literature

For related structures, see: Lee *et al.* (2007 ▶); Matsubara *et al.* (2006 ▶).
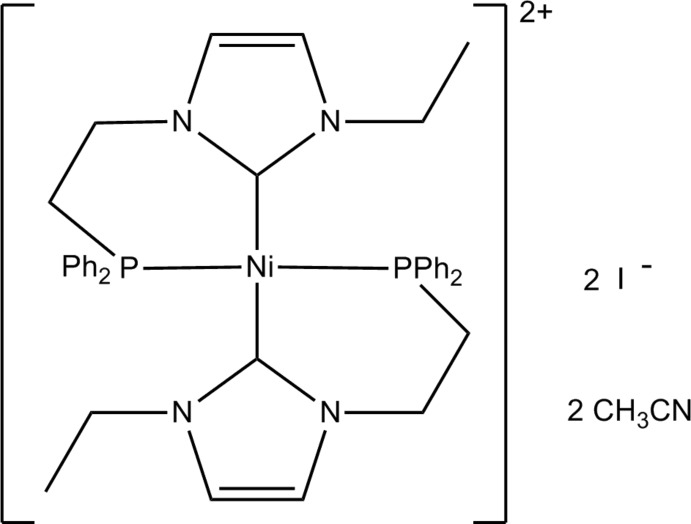


## Experimental

### 

#### Crystal data

[Ni(C_19_H_21_N_2_P)_2_]I_2_·2C_2_H_3_N
*M* *_r_* = 1011.31
Monoclinic, 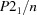

*a* = 11.4453 (4) Å
*b* = 18.7680 (7) Å
*c* = 20.5765 (7) Å
β = 95.900 (1)°
*V* = 4396.5 (3) Å^3^
*Z* = 4
Mo *K*α radiation
μ = 1.95 mm^−1^
*T* = 153 K
0.38 × 0.25 × 0.20 mm

#### Data collection

Bruker SMART APEX diffractometer
Absorption correction: multi-scan (*SADABS*; Sheldrick, 2004 ▶) *T*_min_ = 0.524, *T*_max_ = 0.696
43185 measured reflections
10467 independent reflections
9245 reflections with *I* > 2σ(*I*)
*R*_int_ = 0.028

#### Refinement

*R*[*F* ^2^ > 2σ(*F* ^2^)] = 0.025
*wR*(*F* ^2^) = 0.063
*S* = 1.02
10467 reflections
482 parameters
H-atom parameters constrained
Δρ_max_ = 0.90 e Å^−3^
Δρ_min_ = −0.46 e Å^−3^

### 

Data collection: *SMART* (Bruker, 2002 ▶); cell refinement: *SAINT* (Bruker, 2002 ▶); data reduction: *SAINT*; program(s) used to solve structure: *SHELXTL* (Sheldrick, 2008 ▶); program(s) used to refine structure: *SHELXTL*; molecular graphics: *SHELXTL*; software used to prepare material for publication: *SHELXTL* and local programs.

## Supplementary Material

Crystal structure: contains datablock(s) I, global. DOI: 10.1107/S1600536812028784/bt5955sup1.cif

Structure factors: contains datablock(s) I. DOI: 10.1107/S1600536812028784/bt5955Isup2.hkl

Additional supplementary materials:  crystallographic information; 3D view; checkCIF report

## Figures and Tables

**Fig. 1.**
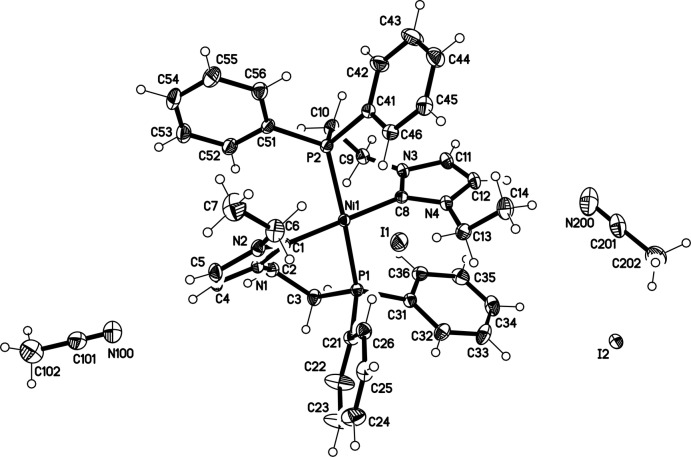
Molecular structure of the title compound. Displacement ellipsoids are drawn at the 50% probability level.

## supplementary crystallographic information

### Experimental

KN(SiMe_3_)_2_ (100 mg, 460 µ-mol) was added to a solution of 3-[2-(diphenylphosphanyl)ethyl]-1-ethylimidazolium-iodide (200 mg, 460 µ-mol) in THF (20 ml). The suspension was stirred at room temperature for 1 h. After removal of KI by filtration [Ni_7_S(S*^t^*Bu)_8_][BzEt_3_N] (166 mg, 153 µ-mol) was added to the filtrate. The color of the resulting suspension changed from pale yellow to black. After the reaction mixture was stirred at room temperature for another 1 h, it was concentrated giving a pale yellow precipitate which was collected by filtration and crystallized by slow evaporation of a concentrated acetonitrile solution to give yellow crystals of Nickel complex.

### Refinement

All Hydrogen atom positions were clearly derived from difference maps, then refined at calculated positions riding on the parent atoms with C—H 0.95 - 0.99 Å and isotropic displacement parameters *U*_iso_(H) = 1.2U_eq_(C) or 1.5U_eq_(–CH_3_). All methyl groups were allowed to rotate but not to tip.

### Crystal data

**Table:**

[Ni(C_19_H_21_N_2_P)_2_]I_2_·2C_2_H_3_N	*F*(000) = 2024
*M**_r_* = 1011.31	*D*_x_ = 1.528 Mg m^−^^3^
Monoclinic, *P*2_1_/*n*	Mo *K*α radiation, λ = 0.71073 Å
Hall symbol: -P 2yn	Cell parameters from 948 reflections
*a* = 11.4453 (4) Å	θ = 2.2–27.6°
*b* = 18.7680 (7) Å	µ = 1.95 mm^−^^1^
*c* = 20.5765 (7) Å	*T* = 153 K
β = 95.900 (1)°	Block, yellow
*V* = 4396.5 (3) Å^3^	0.38 × 0.25 × 0.20 mm
*Z* = 4	

### Data collection

**Table:**

Bruker SMART APEX diffractometer	10467 independent reflections
Radiation source: sealed tube	9245 reflections with *I* > 2σ(*I*)
Graphite monochromator	*R*_int_ = 0.028
φ and ω scans	θ_max_ = 27.9°, θ_min_ = 1.5°
Absorption correction: multi-scan (*SADABS*; Sheldrick, 2004)	*h* = −15→15
*T*_min_ = 0.524, *T*_max_ = 0.696	*k* = −24→24
43185 measured reflections	*l* = −27→24

### Refinement

**Table:**

Refinement on *F*^2^	Primary atom site location: structure-invariant direct methods
Least-squares matrix: full	Secondary atom site location: difference Fourier map
*R*[*F*^2^ > 2σ(*F*^2^)] = 0.025	Hydrogen site location: difference Fourier map
*wR*(*F*^2^) = 0.063	H-atom parameters constrained
*S* = 1.02	*w* = 1/[σ^2^(*F*_o_^2^) + (0.0312*P*)^2^ + 1.6774*P*] where *P* = (*F*_o_^2^ + 2*F*_c_^2^)/3
10467 reflections	(Δ/σ)_max_ = 0.001
482 parameters	Δρ_max_ = 0.90 e Å^−^^3^
0 restraints	Δρ_min_ = −0.46 e Å^−^^3^

### Special details

**Table:**

Geometry. All e.s.d.'s (except the e.s.d. in the dihedral angle between two l.s. planes) are estimated using the full covariance matrix. The cell e.s.d.'s are taken into account individually in the estimation of e.s.d.'s in distances, angles and torsion angles; correlations between e.s.d.'s in cell parameters are only used when they are defined by crystal symmetry. An approximate (isotropic) treatment of cell e.s.d.'s is used for estimating e.s.d.'s involving l.s. planes.
Refinement. Refinement of *F*^2^ against ALL reflections. The weighted *R*-factor *wR* and goodness of fit *S* are based on *F*^2^, conventional *R*-factors *R* are based on *F*, with *F* set to zero for negative *F*^2^. The threshold expression of *F*^2^ > σ(*F*^2^) is used only for calculating *R*-factors(gt) *etc*. and is not relevant to the choice of reflections for refinement. *R*-factors based on *F*^2^ are statistically about twice as large as those based on *F*, and *R*- factors based on ALL data will be even larger.

### Fractional atomic coordinates and isotropic or equivalent isotropic displacement parameters (Å^2^)

**Table:**

	*x*	*y*	*z*	*U*_iso_*/*U*_eq_	
I1	0.224280 (12)	0.296807 (7)	0.139267 (7)	0.03087 (4)	
I2	0.248359 (12)	0.303646 (7)	0.664127 (7)	0.03133 (4)	
Ni1	0.23708 (2)	0.536267 (11)	0.253121 (11)	0.01664 (5)	
P1	0.38866 (4)	0.51971 (2)	0.32586 (2)	0.01820 (9)	
P2	0.08308 (4)	0.58080 (2)	0.19511 (2)	0.01859 (9)	
N1	0.43284 (14)	0.61730 (8)	0.21671 (8)	0.0212 (3)	
N2	0.37969 (15)	0.54375 (8)	0.13943 (8)	0.0241 (3)	
N3	0.05986 (14)	0.56417 (8)	0.33776 (7)	0.0201 (3)	
N4	0.09418 (14)	0.45189 (8)	0.33980 (7)	0.0210 (3)	
C1	0.35249 (16)	0.56602 (9)	0.19843 (9)	0.0200 (4)	
C2	0.43387 (17)	0.65623 (9)	0.27829 (9)	0.0234 (4)	
H2A	0.3570	0.6800	0.2800	0.028*	
H2B	0.4951	0.6936	0.2801	0.028*	
C3	0.45771 (18)	0.60733 (10)	0.33738 (9)	0.0258 (4)	
H3A	0.4286	0.6306	0.3758	0.031*	
H3B	0.5436	0.6009	0.3469	0.031*	
C4	0.50978 (18)	0.62661 (10)	0.17018 (10)	0.0271 (4)	
H4A	0.5737	0.6591	0.1720	0.032*	
C5	0.47650 (19)	0.58065 (11)	0.12179 (10)	0.0296 (4)	
H5A	0.5127	0.5745	0.0826	0.035*	
C6	0.3200 (2)	0.48505 (11)	0.10203 (10)	0.0344 (5)	
H6A	0.2419	0.4776	0.1176	0.041*	
H6B	0.3660	0.4408	0.1109	0.041*	
C7	0.3043 (3)	0.49747 (16)	0.03015 (12)	0.0534 (7)	
H7A	0.2594	0.5414	0.0208	0.080*	
H7B	0.2618	0.4572	0.0085	0.080*	
H7C	0.3814	0.5020	0.0138	0.080*	
C8	0.12495 (16)	0.51368 (9)	0.31233 (8)	0.0184 (3)	
C9	0.06607 (17)	0.63961 (9)	0.31986 (9)	0.0226 (4)	
H9A	0.1471	0.6572	0.3314	0.027*	
H9B	0.0130	0.6675	0.3452	0.027*	
C10	0.03152 (19)	0.65150 (9)	0.24695 (9)	0.0253 (4)	
H10A	0.0642	0.6976	0.2341	0.030*	
H10B	−0.0551	0.6546	0.2391	0.030*	
C11	−0.01031 (18)	0.53514 (10)	0.38181 (9)	0.0257 (4)	
H11A	−0.0635	0.5600	0.4062	0.031*	
C12	0.01178 (18)	0.46453 (10)	0.38319 (10)	0.0264 (4)	
H12A	−0.0227	0.4300	0.4091	0.032*	
C13	0.14367 (18)	0.38162 (10)	0.32701 (10)	0.0276 (4)	
H13A	0.2040	0.3693	0.3632	0.033*	
H13B	0.1828	0.3840	0.2863	0.033*	
C14	0.0519 (2)	0.32365 (11)	0.32022 (12)	0.0356 (5)	
H14A	0.0167	0.3186	0.3614	0.053*	
H14B	0.0886	0.2785	0.3096	0.053*	
H14C	−0.0093	0.3362	0.2852	0.053*	
C21	0.49744 (17)	0.45793 (10)	0.30145 (9)	0.0236 (4)	
C22	0.6142 (2)	0.46212 (15)	0.32602 (12)	0.0483 (7)	
H22A	0.6410	0.5014	0.3526	0.058*	
C23	0.6925 (2)	0.40908 (17)	0.31204 (14)	0.0600 (9)	
H23A	0.7724	0.4120	0.3296	0.072*	
C24	0.6552 (2)	0.35258 (13)	0.27305 (12)	0.0426 (6)	
H24A	0.7089	0.3161	0.2642	0.051*	
C25	0.5404 (2)	0.34869 (11)	0.24691 (11)	0.0324 (5)	
H25A	0.5150	0.3101	0.2191	0.039*	
C26	0.46067 (18)	0.40106 (10)	0.26090 (10)	0.0260 (4)	
H26A	0.3811	0.3980	0.2428	0.031*	
C31	0.36241 (17)	0.49181 (10)	0.40768 (9)	0.0212 (4)	
C32	0.40165 (19)	0.42627 (10)	0.43330 (10)	0.0289 (4)	
H32A	0.4522	0.3977	0.4103	0.035*	
C33	0.3670 (2)	0.40258 (12)	0.49236 (10)	0.0361 (5)	
H33A	0.3944	0.3580	0.5098	0.043*	
C34	0.2930 (2)	0.44365 (13)	0.52567 (10)	0.0377 (5)	
H34A	0.2669	0.4265	0.5651	0.045*	
C35	0.2566 (2)	0.50980 (13)	0.50185 (10)	0.0343 (5)	
H35A	0.2079	0.5386	0.5258	0.041*	
C36	0.29119 (18)	0.53406 (11)	0.44316 (9)	0.0265 (4)	
H36A	0.2663	0.5796	0.4270	0.032*	
C41	−0.03503 (16)	0.51692 (9)	0.17878 (9)	0.0208 (4)	
C42	−0.15305 (19)	0.53628 (11)	0.17275 (12)	0.0355 (5)	
H42A	−0.1751	0.5845	0.1783	0.043*	
C43	−0.2386 (2)	0.48414 (13)	0.15855 (14)	0.0458 (6)	
H43A	−0.3193	0.4971	0.1541	0.055*	
C44	−0.2074 (2)	0.41393 (12)	0.15091 (11)	0.0369 (5)	
H44A	−0.2665	0.3788	0.1415	0.044*	
C45	−0.09055 (19)	0.39452 (11)	0.15685 (10)	0.0285 (4)	
H45A	−0.0691	0.3462	0.1514	0.034*	
C46	−0.00461 (17)	0.44588 (10)	0.17074 (9)	0.0240 (4)	
H46A	0.0759	0.4325	0.1748	0.029*	
C51	0.09739 (17)	0.62542 (9)	0.11791 (9)	0.0221 (4)	
C52	0.18174 (19)	0.67909 (10)	0.11592 (10)	0.0279 (4)	
H52A	0.2287	0.6924	0.1548	0.034*	
C53	0.1970 (2)	0.71293 (11)	0.05741 (11)	0.0333 (5)	
H53A	0.2544	0.7494	0.0564	0.040*	
C54	0.1291 (2)	0.69380 (11)	0.00050 (11)	0.0357 (5)	
H54A	0.1404	0.7168	−0.0395	0.043*	
C55	0.0449 (2)	0.64114 (12)	0.00208 (10)	0.0370 (5)	
H55A	−0.0025	0.6285	−0.0368	0.044*	
C56	0.02917 (19)	0.60646 (11)	0.06056 (10)	0.0286 (4)	
H56A	−0.0281	0.5699	0.0612	0.034*	
N100	0.7173 (2)	0.67617 (17)	0.08385 (15)	0.0795 (9)	
C101	0.7765 (2)	0.72052 (16)	0.06886 (14)	0.0500 (7)	
C102	0.8519 (3)	0.77672 (15)	0.04768 (16)	0.0550 (7)	
H10C	0.8576	0.7722	0.0007	0.082*	
H10D	0.9304	0.7725	0.0713	0.082*	
H10E	0.8185	0.8233	0.0568	0.082*	
N200	−0.0568 (3)	0.33739 (14)	0.49174 (12)	0.0646 (7)	
C201	−0.0278 (2)	0.28314 (14)	0.51184 (12)	0.0428 (6)	
C202	0.0079 (2)	0.21328 (13)	0.53768 (14)	0.0477 (6)	
H20A	−0.0554	0.1929	0.5605	0.072*	
H20B	0.0789	0.2181	0.5683	0.072*	
H20C	0.0241	0.1818	0.5017	0.072*	

### Atomic displacement parameters (Å^2^)

**Table:**

	*U*^11^	*U*^22^	*U*^33^	*U*^12^	*U*^13^	*U*^23^
I1	0.03160 (8)	0.02166 (7)	0.04030 (9)	0.00370 (5)	0.00828 (6)	0.00471 (5)
I2	0.02307 (8)	0.02738 (7)	0.04424 (9)	−0.00132 (5)	0.00681 (6)	0.00706 (5)
Ni1	0.01732 (12)	0.01732 (11)	0.01559 (11)	−0.00023 (8)	0.00317 (8)	0.00100 (8)
P1	0.0191 (2)	0.0190 (2)	0.0167 (2)	−0.00021 (17)	0.00253 (17)	0.00000 (16)
P2	0.0201 (2)	0.0173 (2)	0.0185 (2)	0.00086 (17)	0.00260 (18)	0.00147 (16)
N1	0.0199 (8)	0.0211 (7)	0.0233 (8)	0.0010 (6)	0.0052 (6)	0.0040 (6)
N2	0.0294 (9)	0.0227 (8)	0.0213 (8)	0.0025 (6)	0.0076 (7)	0.0016 (6)
N3	0.0210 (8)	0.0212 (7)	0.0187 (7)	−0.0034 (6)	0.0052 (6)	−0.0015 (6)
N4	0.0216 (8)	0.0200 (7)	0.0218 (8)	−0.0029 (6)	0.0038 (6)	0.0016 (6)
C1	0.0216 (9)	0.0200 (8)	0.0190 (9)	0.0034 (7)	0.0043 (7)	0.0030 (7)
C2	0.0253 (10)	0.0185 (8)	0.0263 (10)	−0.0012 (7)	0.0019 (8)	−0.0001 (7)
C3	0.0295 (11)	0.0238 (9)	0.0236 (10)	−0.0072 (8)	0.0006 (8)	0.0001 (7)
C4	0.0248 (10)	0.0262 (9)	0.0322 (11)	0.0012 (8)	0.0125 (8)	0.0081 (8)
C5	0.0323 (11)	0.0295 (10)	0.0295 (11)	0.0046 (8)	0.0159 (9)	0.0065 (8)
C6	0.0480 (14)	0.0288 (10)	0.0271 (11)	−0.0033 (9)	0.0081 (9)	−0.0074 (8)
C7	0.068 (2)	0.0629 (17)	0.0301 (13)	−0.0091 (14)	0.0081 (12)	−0.0096 (12)
C8	0.0175 (9)	0.0201 (8)	0.0172 (8)	−0.0022 (6)	−0.0008 (7)	0.0002 (6)
C9	0.0262 (10)	0.0166 (8)	0.0260 (10)	−0.0012 (7)	0.0072 (8)	−0.0037 (7)
C10	0.0329 (11)	0.0180 (8)	0.0258 (10)	0.0051 (7)	0.0068 (8)	0.0001 (7)
C11	0.0255 (10)	0.0295 (10)	0.0237 (10)	−0.0042 (8)	0.0097 (8)	−0.0011 (7)
C12	0.0261 (10)	0.0288 (10)	0.0256 (10)	−0.0060 (8)	0.0087 (8)	0.0029 (8)
C13	0.0284 (11)	0.0195 (9)	0.0347 (11)	0.0003 (7)	0.0030 (8)	0.0019 (8)
C14	0.0455 (14)	0.0226 (10)	0.0395 (12)	−0.0084 (9)	0.0083 (10)	−0.0037 (9)
C21	0.0229 (10)	0.0287 (9)	0.0196 (9)	0.0045 (7)	0.0047 (7)	−0.0012 (7)
C22	0.0282 (12)	0.0676 (17)	0.0467 (14)	0.0130 (11)	−0.0070 (11)	−0.0356 (13)
C23	0.0275 (13)	0.091 (2)	0.0582 (17)	0.0243 (13)	−0.0094 (12)	−0.0367 (16)
C24	0.0423 (14)	0.0488 (14)	0.0379 (13)	0.0208 (11)	0.0095 (11)	−0.0073 (10)
C25	0.0420 (13)	0.0249 (10)	0.0324 (11)	0.0018 (9)	0.0136 (10)	−0.0016 (8)
C26	0.0272 (10)	0.0224 (9)	0.0290 (10)	−0.0017 (7)	0.0056 (8)	−0.0005 (7)
C31	0.0225 (9)	0.0246 (9)	0.0162 (8)	−0.0034 (7)	0.0002 (7)	0.0005 (7)
C32	0.0370 (12)	0.0253 (9)	0.0236 (10)	0.0006 (8)	−0.0009 (8)	0.0002 (8)
C33	0.0533 (15)	0.0294 (10)	0.0238 (10)	−0.0079 (10)	−0.0051 (10)	0.0068 (8)
C34	0.0416 (13)	0.0530 (14)	0.0183 (10)	−0.0182 (11)	0.0013 (9)	0.0040 (9)
C35	0.0290 (11)	0.0533 (14)	0.0209 (10)	−0.0009 (10)	0.0043 (8)	−0.0036 (9)
C36	0.0263 (10)	0.0327 (10)	0.0202 (9)	0.0029 (8)	0.0012 (8)	−0.0007 (7)
C41	0.0207 (9)	0.0218 (8)	0.0199 (9)	−0.0007 (7)	0.0023 (7)	0.0019 (7)
C42	0.0236 (11)	0.0288 (10)	0.0540 (14)	0.0027 (8)	0.0032 (10)	0.0035 (10)
C43	0.0217 (11)	0.0438 (13)	0.0713 (18)	−0.0018 (10)	0.0017 (11)	0.0017 (12)
C44	0.0327 (12)	0.0378 (12)	0.0395 (12)	−0.0126 (9)	0.0008 (10)	−0.0007 (10)
C45	0.0350 (12)	0.0264 (10)	0.0240 (10)	−0.0058 (8)	0.0024 (8)	−0.0020 (8)
C46	0.0230 (10)	0.0264 (9)	0.0225 (9)	0.0007 (7)	0.0022 (7)	−0.0015 (7)
C51	0.0244 (10)	0.0212 (8)	0.0211 (9)	0.0047 (7)	0.0044 (7)	0.0049 (7)
C52	0.0314 (11)	0.0245 (9)	0.0273 (10)	−0.0012 (8)	0.0002 (8)	0.0042 (8)
C53	0.0383 (13)	0.0266 (10)	0.0364 (12)	−0.0016 (9)	0.0097 (10)	0.0079 (8)
C54	0.0490 (15)	0.0339 (11)	0.0261 (11)	0.0047 (10)	0.0124 (10)	0.0102 (8)
C55	0.0475 (14)	0.0428 (12)	0.0204 (10)	0.0018 (10)	0.0024 (9)	0.0031 (9)
C56	0.0319 (11)	0.0309 (10)	0.0233 (10)	−0.0016 (8)	0.0038 (8)	0.0013 (8)
N100	0.0528 (17)	0.100 (2)	0.090 (2)	0.0178 (16)	0.0270 (15)	0.0565 (18)
C101	0.0398 (15)	0.0664 (18)	0.0453 (15)	0.0161 (13)	0.0115 (12)	0.0168 (13)
C102	0.0548 (18)	0.0464 (15)	0.0640 (18)	−0.0037 (13)	0.0079 (14)	−0.0065 (13)
N200	0.088 (2)	0.0563 (15)	0.0491 (14)	−0.0249 (14)	0.0045 (13)	0.0085 (12)
C201	0.0525 (16)	0.0455 (14)	0.0316 (12)	−0.0189 (12)	0.0098 (11)	−0.0023 (10)
C202	0.0454 (16)	0.0466 (14)	0.0512 (16)	−0.0059 (11)	0.0052 (12)	−0.0060 (12)

### Geometric parameters (Å, º)

**Table:**

Ni1—C1	1.9049 (18)	C22—C23	1.389 (3)
Ni1—C8	1.9059 (18)	C22—H22A	0.9500
Ni1—P2	2.1904 (5)	C23—C24	1.371 (4)
Ni1—P1	2.1938 (5)	C23—H23A	0.9500
P1—C21	1.8098 (19)	C24—C25	1.370 (3)
P1—C31	1.8174 (18)	C24—H24A	0.9500
P1—C3	1.8292 (19)	C25—C26	1.391 (3)
P2—C41	1.8124 (19)	C25—H25A	0.9500
P2—C51	1.8178 (18)	C26—H26A	0.9500
P2—C10	1.8374 (19)	C31—C32	1.394 (3)
N1—C1	1.357 (2)	C31—C36	1.396 (3)
N1—C4	1.377 (2)	C32—C33	1.389 (3)
N1—C2	1.462 (2)	C32—H32A	0.9500
N2—C1	1.350 (2)	C33—C34	1.379 (3)
N2—C5	1.386 (3)	C33—H33A	0.9500
N2—C6	1.471 (3)	C34—C35	1.383 (3)
N3—C8	1.344 (2)	C34—H34A	0.9500
N3—C11	1.383 (2)	C35—C36	1.386 (3)
N3—C9	1.466 (2)	C35—H35A	0.9500
N4—C8	1.352 (2)	C36—H36A	0.9500
N4—C12	1.384 (2)	C41—C42	1.392 (3)
N4—C13	1.470 (2)	C41—C46	1.392 (3)
C2—C3	1.526 (3)	C42—C43	1.394 (3)
C2—H2A	0.9900	C42—H42A	0.9500
C2—H2B	0.9900	C43—C44	1.379 (3)
C3—H3A	0.9900	C43—H43A	0.9500
C3—H3B	0.9900	C44—C45	1.379 (3)
C4—C5	1.343 (3)	C44—H44A	0.9500
C4—H4A	0.9500	C45—C46	1.386 (3)
C5—H5A	0.9500	C45—H45A	0.9500
C6—C7	1.490 (3)	C46—H46A	0.9500
C6—H6A	0.9900	C51—C56	1.393 (3)
C6—H6B	0.9900	C51—C52	1.399 (3)
C7—H7A	0.9800	C52—C53	1.388 (3)
C7—H7B	0.9800	C52—H52A	0.9500
C7—H7C	0.9800	C53—C54	1.385 (3)
C9—C10	1.528 (3)	C53—H53A	0.9500
C9—H9A	0.9900	C54—C55	1.383 (3)
C9—H9B	0.9900	C54—H54A	0.9500
C10—H10A	0.9900	C55—C56	1.396 (3)
C10—H10B	0.9900	C55—H55A	0.9500
C11—C12	1.349 (3)	C56—H56A	0.9500
C11—H11A	0.9500	N100—C101	1.135 (4)
C12—H12A	0.9500	C101—C102	1.458 (4)
C13—C14	1.509 (3)	C102—H10C	0.9800
C13—H13A	0.9900	C102—H10D	0.9800
C13—H13B	0.9900	C102—H10E	0.9800
C14—H14A	0.9800	N200—C201	1.136 (4)
C14—H14B	0.9800	C201—C202	1.457 (4)
C14—H14C	0.9800	C202—H20A	0.9800
C21—C22	1.381 (3)	C202—H20B	0.9800
C21—C26	1.393 (3)	C202—H20C	0.9800
			
C1—Ni1—C8	175.02 (8)	C13—C14—H14B	109.5
C1—Ni1—P2	97.71 (6)	H14A—C14—H14B	109.5
C8—Ni1—P2	82.89 (5)	C13—C14—H14C	109.5
C1—Ni1—P1	84.08 (6)	H14A—C14—H14C	109.5
C8—Ni1—P1	94.00 (5)	H14B—C14—H14C	109.5
P2—Ni1—P1	164.05 (2)	C22—C21—C26	118.96 (18)
C21—P1—C31	104.85 (9)	C22—C21—P1	121.94 (15)
C21—P1—C3	108.16 (9)	C26—C21—P1	118.89 (15)
C31—P1—C3	104.44 (9)	C21—C22—C23	120.2 (2)
C21—P1—Ni1	114.82 (6)	C21—C22—H22A	119.9
C31—P1—Ni1	118.58 (6)	C23—C22—H22A	119.9
C3—P1—Ni1	105.18 (7)	C24—C23—C22	120.4 (2)
C41—P2—C51	105.97 (9)	C24—C23—H23A	119.8
C41—P2—C10	107.90 (9)	C22—C23—H23A	119.8
C51—P2—C10	103.76 (8)	C25—C24—C23	120.0 (2)
C41—P2—Ni1	113.06 (6)	C25—C24—H24A	120.0
C51—P2—Ni1	120.82 (6)	C23—C24—H24A	120.0
C10—P2—Ni1	104.34 (7)	C24—C25—C26	120.3 (2)
C1—N1—C4	111.15 (16)	C24—C25—H25A	119.9
C1—N1—C2	122.66 (15)	C26—C25—H25A	119.9
C4—N1—C2	126.20 (16)	C25—C26—C21	120.1 (2)
C1—N2—C5	110.19 (16)	C25—C26—H26A	120.0
C1—N2—C6	124.37 (17)	C21—C26—H26A	120.0
C5—N2—C6	125.29 (17)	C32—C31—C36	119.09 (18)
C8—N3—C11	111.09 (15)	C32—C31—P1	121.80 (15)
C8—N3—C9	122.66 (15)	C36—C31—P1	118.80 (14)
C11—N3—C9	126.25 (15)	C33—C32—C31	120.2 (2)
C8—N4—C12	110.28 (15)	C33—C32—H32A	119.9
C8—N4—C13	124.95 (16)	C31—C32—H32A	119.9
C12—N4—C13	124.75 (16)	C34—C33—C32	120.1 (2)
N2—C1—N1	104.85 (15)	C34—C33—H33A	120.0
N2—C1—Ni1	132.73 (14)	C32—C33—H33A	120.0
N1—C1—Ni1	122.36 (13)	C33—C34—C35	120.2 (2)
N1—C2—C3	112.02 (15)	C33—C34—H34A	119.9
N1—C2—H2A	109.2	C35—C34—H34A	119.9
C3—C2—H2A	109.2	C34—C35—C36	120.1 (2)
N1—C2—H2B	109.2	C34—C35—H35A	120.0
C3—C2—H2B	109.2	C36—C35—H35A	120.0
H2A—C2—H2B	107.9	C35—C36—C31	120.23 (19)
C2—C3—P1	113.46 (13)	C35—C36—H36A	119.9
C2—C3—H3A	108.9	C31—C36—H36A	119.9
P1—C3—H3A	108.9	C42—C41—C46	119.45 (18)
C2—C3—H3B	108.9	C42—C41—P2	122.84 (15)
P1—C3—H3B	108.9	C46—C41—P2	117.69 (14)
H3A—C3—H3B	107.7	C41—C42—C43	119.3 (2)
C5—C4—N1	106.33 (17)	C41—C42—H42A	120.3
C5—C4—H4A	126.8	C43—C42—H42A	120.3
N1—C4—H4A	126.8	C44—C43—C42	120.6 (2)
C4—C5—N2	107.47 (17)	C44—C43—H43A	119.7
C4—C5—H5A	126.3	C42—C43—H43A	119.7
N2—C5—H5A	126.3	C43—C44—C45	120.2 (2)
N2—C6—C7	113.84 (19)	C43—C44—H44A	119.9
N2—C6—H6A	108.8	C45—C44—H44A	119.9
C7—C6—H6A	108.8	C44—C45—C46	119.72 (19)
N2—C6—H6B	108.8	C44—C45—H45A	120.1
C7—C6—H6B	108.8	C46—C45—H45A	120.1
H6A—C6—H6B	107.7	C45—C46—C41	120.64 (19)
C6—C7—H7A	109.5	C45—C46—H46A	119.7
C6—C7—H7B	109.5	C41—C46—H46A	119.7
H7A—C7—H7B	109.5	C56—C51—C52	119.18 (18)
C6—C7—H7C	109.5	C56—C51—P2	121.99 (15)
H7A—C7—H7C	109.5	C52—C51—P2	118.80 (15)
H7B—C7—H7C	109.5	C53—C52—C51	120.26 (19)
N3—C8—N4	105.26 (15)	C53—C52—H52A	119.9
N3—C8—Ni1	121.91 (13)	C51—C52—H52A	119.9
N4—C8—Ni1	132.78 (14)	C54—C53—C52	120.3 (2)
N3—C9—C10	111.92 (14)	C54—C53—H53A	119.8
N3—C9—H9A	109.2	C52—C53—H53A	119.8
C10—C9—H9A	109.2	C55—C54—C53	119.8 (2)
N3—C9—H9B	109.2	C55—C54—H54A	120.1
C10—C9—H9B	109.2	C53—C54—H54A	120.1
H9A—C9—H9B	107.9	C54—C55—C56	120.4 (2)
C9—C10—P2	113.48 (13)	C54—C55—H55A	119.8
C9—C10—H10A	108.9	C56—C55—H55A	119.8
P2—C10—H10A	108.9	C51—C56—C55	120.0 (2)
C9—C10—H10B	108.9	C51—C56—H56A	120.0
P2—C10—H10B	108.9	C55—C56—H56A	120.0
H10A—C10—H10B	107.7	N100—C101—C102	178.3 (4)
C12—C11—N3	106.30 (17)	C101—C102—H10C	109.5
C12—C11—H11A	126.9	C101—C102—H10D	109.5
N3—C11—H11A	126.8	H10C—C102—H10D	109.5
C11—C12—N4	107.06 (16)	C101—C102—H10E	109.5
C11—C12—H12A	126.5	H10C—C102—H10E	109.5
N4—C12—H12A	126.5	H10D—C102—H10E	109.5
N4—C13—C14	112.71 (17)	N200—C201—C202	179.2 (3)
N4—C13—H13A	109.0	C201—C202—H20A	109.5
C14—C13—H13A	109.0	C201—C202—H20B	109.5
N4—C13—H13B	109.0	H20A—C202—H20B	109.5
C14—C13—H13B	109.0	C201—C202—H20C	109.5
H13A—C13—H13B	107.8	H20A—C202—H20C	109.5
C13—C14—H14A	109.5	H20B—C202—H20C	109.5
			
C1—Ni1—P1—C21	−58.52 (9)	C9—N3—C11—C12	179.95 (18)
C8—Ni1—P1—C21	126.08 (9)	N3—C11—C12—N4	−0.4 (2)
P2—Ni1—P1—C21	−155.87 (9)	C8—N4—C12—C11	1.0 (2)
C1—Ni1—P1—C31	176.48 (9)	C13—N4—C12—C11	179.26 (18)
C8—Ni1—P1—C31	1.08 (9)	C8—N4—C13—C14	−138.59 (19)
P2—Ni1—P1—C31	79.13 (10)	C12—N4—C13—C14	43.4 (3)
C1—Ni1—P1—C3	60.24 (9)	C31—P1—C21—C22	−75.0 (2)
C8—Ni1—P1—C3	−115.15 (9)	C3—P1—C21—C22	36.0 (2)
P2—Ni1—P1—C3	−37.10 (11)	Ni1—P1—C21—C22	153.10 (19)
C1—Ni1—P2—C41	129.59 (9)	C31—P1—C21—C26	99.64 (16)
C8—Ni1—P2—C41	−55.39 (8)	C3—P1—C21—C26	−149.36 (15)
P1—Ni1—P2—C41	−134.97 (9)	Ni1—P1—C21—C26	−32.27 (17)
C1—Ni1—P2—C51	2.57 (9)	C26—C21—C22—C23	−2.0 (4)
C8—Ni1—P2—C51	177.59 (9)	P1—C21—C22—C23	172.6 (2)
P1—Ni1—P2—C51	98.01 (10)	C21—C22—C23—C24	0.9 (5)
C1—Ni1—P2—C10	−113.43 (9)	C22—C23—C24—C25	0.9 (5)
C8—Ni1—P2—C10	61.59 (8)	C23—C24—C25—C26	−1.4 (4)
P1—Ni1—P2—C10	−17.99 (11)	C24—C25—C26—C21	0.2 (3)
C5—N2—C1—N1	0.4 (2)	C22—C21—C26—C25	1.5 (3)
C6—N2—C1—N1	176.23 (18)	P1—C21—C26—C25	−173.29 (15)
C5—N2—C1—Ni1	−176.74 (15)	C21—P1—C31—C32	−13.28 (19)
C6—N2—C1—Ni1	−0.9 (3)	C3—P1—C31—C32	−126.93 (17)
C4—N1—C1—N2	−0.5 (2)	Ni1—P1—C31—C32	116.44 (16)
C2—N1—C1—N2	179.40 (15)	C21—P1—C31—C36	173.14 (15)
C4—N1—C1—Ni1	177.04 (13)	C3—P1—C31—C36	59.49 (17)
C2—N1—C1—Ni1	−3.1 (2)	Ni1—P1—C31—C36	−57.14 (17)
P2—Ni1—C1—N2	−72.74 (18)	C36—C31—C32—C33	2.0 (3)
P1—Ni1—C1—N2	123.22 (18)	P1—C31—C32—C33	−171.53 (16)
P2—Ni1—C1—N1	110.48 (14)	C31—C32—C33—C34	0.4 (3)
P1—Ni1—C1—N1	−53.55 (14)	C32—C33—C34—C35	−2.6 (3)
C1—N1—C2—C3	63.6 (2)	C33—C34—C35—C36	2.2 (3)
C4—N1—C2—C3	−116.5 (2)	C34—C35—C36—C31	0.3 (3)
N1—C2—C3—P1	−37.4 (2)	C32—C31—C36—C35	−2.4 (3)
C21—P1—C3—C2	97.39 (16)	P1—C31—C36—C35	171.38 (16)
C31—P1—C3—C2	−151.33 (14)	C51—P2—C41—C42	−77.59 (19)
Ni1—P1—C3—C2	−25.75 (16)	C10—P2—C41—C42	33.0 (2)
C1—N1—C4—C5	0.4 (2)	Ni1—P2—C41—C42	147.91 (16)
C2—N1—C4—C5	−179.53 (17)	C51—P2—C41—C46	101.06 (15)
N1—C4—C5—N2	−0.1 (2)	C10—P2—C41—C46	−148.30 (15)
C1—N2—C5—C4	−0.2 (2)	Ni1—P2—C41—C46	−33.44 (16)
C6—N2—C5—C4	−175.97 (19)	C46—C41—C42—C43	−0.2 (3)
C1—N2—C6—C7	142.7 (2)	P2—C41—C42—C43	178.42 (19)
C5—N2—C6—C7	−42.2 (3)	C41—C42—C43—C44	0.4 (4)
C11—N3—C8—N4	1.0 (2)	C42—C43—C44—C45	−0.4 (4)
C9—N3—C8—N4	−179.35 (16)	C43—C44—C45—C46	0.2 (3)
C11—N3—C8—Ni1	−176.86 (13)	C44—C45—C46—C41	0.0 (3)
C9—N3—C8—Ni1	2.8 (2)	C42—C41—C46—C45	0.0 (3)
C12—N4—C8—N3	−1.2 (2)	P2—C41—C46—C45	−178.70 (15)
C13—N4—C8—N3	−179.47 (17)	C41—P2—C51—C56	−2.76 (19)
C12—N4—C8—Ni1	176.30 (15)	C10—P2—C51—C56	−116.29 (17)
C13—N4—C8—Ni1	−2.0 (3)	Ni1—P2—C51—C56	127.42 (15)
P2—Ni1—C8—N3	−58.63 (14)	C41—P2—C51—C52	178.98 (15)
P1—Ni1—C8—N3	105.65 (14)	C10—P2—C51—C52	65.45 (17)
P2—Ni1—C8—N4	124.22 (18)	Ni1—P2—C51—C52	−50.85 (17)
P1—Ni1—C8—N4	−71.50 (17)	C56—C51—C52—C53	0.0 (3)
C8—N3—C9—C10	60.6 (2)	P2—C51—C52—C53	178.31 (16)
C11—N3—C9—C10	−119.7 (2)	C51—C52—C53—C54	−0.1 (3)
N3—C9—C10—P2	−37.7 (2)	C52—C53—C54—C55	0.6 (3)
C41—P2—C10—C9	94.98 (15)	C53—C54—C55—C56	−1.0 (3)
C51—P2—C10—C9	−152.88 (14)	C52—C51—C56—C55	−0.4 (3)
Ni1—P2—C10—C9	−25.51 (15)	P2—C51—C56—C55	−178.62 (16)
C8—N3—C11—C12	−0.4 (2)	C54—C55—C56—C51	0.8 (3)
